# A multi-centre cohort study of short term outcomes of hospital treatment for anorexia nervosa in the UK

**DOI:** 10.1186/1471-244X-13-287

**Published:** 2013-11-07

**Authors:** Elizabeth Goddard, Rebecca Hibbs, Simone Raenker, Laura Salerno, Jon Arcelus, Nicky Boughton, Frances Connan, Ken Goss, Bert Laszlo, John Morgan, Kim Moore, David Robertson, Saeidi S, Christa Schreiber-Kounine, Sonu Sharma, Linette Whitehead, Ulrike Schmidt, Janet Treasure

**Affiliations:** 1 Department of Psychological Medicine, Section of Eating Disorders, King’s College London, Institute of Psychiatry, London, UK; 2 Department of Psychology, University of Palermo, Palermo, Italy; 3 Eating Disorders Service, Brandon Unit, Leicestershire Partnership NHS Trust, Leicester, UK; 4 Cotswold House Eating Disorders Service, Oxford Health NHS Foundation Trust, Oxford, UK; 5 Vincent Square Eating Disorders Service, Central and North West London NHS Foundation Trust, London, UK; 6 Eating Disorders Service, Coventry and Warwickshire NHS Partnership Trust, Coventry, UK; 7 Wonford House Hospital, Devon Partnership NHS Trust, Exeter, UK; 8 Yorkshire Centre for Eating Disorders, Leeds and St George’s University of London, Leeds, UK; 9 Kinver Centre, Eating Disorders, South Staffordshire and Shropshire NHS Foundation Trust, Staffordshire, UK; 10 National Centre for Mental Health, Birmingham and Solihull Mental Health NHS Foundation Trust, Birmingham, UK; 11 STEPS Eating Disorders Unit, Avon and Wiltshire Partnership Mental Health NHS Trust, Bristol, UK; 12 Eating Disorders Service, The Priory Hospital Cheadle Royal, Manchester, UK

**Keywords:** Eating disorders, Anorexia Nervosa, Inpatients, Treatment response, Predictors, Treatment

## Abstract

**Background:**

Individual, family and service level characteristics and outcomes are described for adult and adolescent patients receiving specialist inpatient or day patient treatment for anorexia nervosa (AN). Potential predictors of treatment outcome are explored.

**Method:**

Admission and discharge data were collected from patients admitted at 14 UK hospital treatment units for AN over a period of three years (adult units N = 12; adolescent N = 2) (patients N = 177).

**Results:**

One hundred and seventy-seven patients with a severe and enduring illness with wide functional impairment took part in the study. Following inpatient care, physical improvement was moderate/good with a large increase in BMI, although most patients continued to have a clinical level of eating disorder symptoms at discharge. The potentially modifiable predictors of outcome included confidence to change, social functioning and carer expressed emotion and control.

**Conclusions:**

Overall, the response to inpatient treatment was modest particularly in the group with a severe enduring form of illness. Adolescents had a better response. Although inpatient treatment produces an improvement in physical health there was less improvement in other eating disorder and mood symptoms. As predicted by the carer interpersonal maintenance model, carer behaviour may influence the response to inpatient care, as may improved social functioning and confidence to change.

## Background

There is a large amount of uncertainty about the management of patients with severe, persisting eating disorders (EDs) [1,2]. In part, this is reflected in the cultural and temporal changes in the use of inpatient treatment and the variation in service level treatment parameters in Europe [3] and in the USA and Canada [4]. More recently, day patient services (typically a 9–5pm programme, 5 days a week with 2 or 3 meals) are being offered as an alternative to inpatient care (residential, 7 days a week with all meals), including in the UK [5] for those not at high medical risk [6-10]. A recent meta-analysis, which examined factors that predicted weight gain in anorexia nervosa (AN), concluded that inpatient care was associated with the fastest change [11]. An additional goal of inpatient care is to facilitate a move towards recovery and a better quality of life. Therefore broader factors, other than weight gain alone, have to be considered.

In the UK the National Institute of Clinical Excellence (NICE) published guidelines for the treatment of AN and recommend inpatient treatment for those with high medical and/or psychosocial risk and who have failed to respond to less intensive treatments [12]. In line with these guidelines, current cohorts are older and have a more severe and enduring illness [13,14] than in previous UK inpatient cohorts from 1959–64 [15] and 1979–84 [16]. Studies describing the clinical status and treatment response of patients admitted for hospital care in the UK are usually based on single centre studies and therefore do not capture variation in case mix across sites. Other countries have different service models of care than those outlined in the NICE guidelines, but there is a lack of evidence upon which to base the models of care provision.

The restoration of nutritional health in patients with this degree of severe weight loss takes time and this explains why, compared to other psychiatric disorders, AN has the highest proportion of admissions in the UK with an average length of stay over 90 days (26.8%) and the longest median length of stay (36 days) [17]. This contributes to the high cost of illness [18,19]. Furthermore, poor treatment adherence (drop out or failure to meet treatment goals) is a feature of inpatient care [20,21]. Identifying factors that can be targeted to increase the cost-effectiveness and acceptability of inpatient care has implications for the individual, their family and service providers. The main factors that predict outcome from inpatient care are fixed and include the severity (e.g. BMI) and duration of illness [5,22]. However, both intra- and inter-personal psychosocial factors can be modified and may influence the response to treatment. For example, individual factors such as motivation to change have been associated with reduced rates of relapse post-inpatient care and may be important for engendering change within the hospital setting [23].

Interpersonal factors form a key component of a theoretical model designed to explain treatment resistance in AN [24]. For example. in response to the manifestations of AN in the patient, carers exhibit measurable anxiety and depression and it is hypothesised that these symptoms directly and indirectly affect the individual with AN, [24,25]. Moreover, eating symptoms and distress have been shown to be associated with carer distress, expressed emotion, and psychological control in a model developed using cross sectional data [26]. It is therefore of interest to examine whether these factors have an impact on response to inpatient treatment.

### The present study

The first aim of this paper is to describe the clinical features of patients admitted to a cross section of UK services for inpatient and day patient care on admission and at discharge. A second aim is to examine whether intra- (ED symptoms, mood, and motivation to change) and interpersonal factors (perceived expressed emotion, control, quality of social relationships) contribute to the response to inpatient care in terms of ED psychopathology. It is our expectation that ED symptoms, low mood and low motivation to change will contribute to a more negative response to inpatient care. Similarly, it is our expectation that high perceived carer expressed emotion and control and low quality of social relationships will contribute negatively to the response to inpatient care.

## Method

### Participants

Participants were recruited from a consecutive series of inpatients and day patients admitted to specialist ED hospital services in the UK over a three year period (September 2008 and August 2011). Day patients in this context were patients who required non-residential intensive specialist treatment (≥ 4 days a week). The families of these patients agreed to participate in a randomised controlled trial (RCT) to examine the impact of a guided self-help skills training intervention on the outcomes of carers and the individual with AN post discharge from hospital treatment. However, only patient data are included in this paper. Trial protocol and recruitment procedure are described in more detail in another paper [27]. The inclusion criteria required individuals to be fluent in English, have a primary diagnosis of AN or Eating Disorder Not Otherwise Specified with Anorexic symptoms (diagnosed using clinical interview ICD 10 criteria), and have at least one carer consent to participate in the project. Some hospitals used a step down procedure whereby patients may be gradually moved to a less intensive form of treatment (i.e. from inpatient to day patient) prior to discharge into outpatient services or the community. Patients were considered discharged when they ceased to receive intensive treatment for their ED (that is, inpatient treatment or day patient treatment ≥ 4 days a week). Those who were transferred to residential care or day care ≥ 4 days a week where they continued to receive specialist and intensive ED treatment were not considered to be discharged; however, those who moved on to more generic supported housing services where they did not receive specialist intensive support were. Patients had to maintain their “discharged” status for a minimum of four weeks to be counted as formally discharged. If they returned to hospital within four weeks, this period was considered part of a continuous hospital admission. Details on whether patients completed the treatment programme or discharged themselves without recommendation by the treatment centre were not available for all sites and are therefore not included.

### Participating centres

Data from participants of 14 hospitals were included in this paper. Twelve hospitals are adult ED specialist inpatient units and two are adolescent hospital units (one specialist ED and one general psychiatric with ED beds). Four sites recruited inpatients as well as day patients into the study. We wanted to have an ecologically relevant sample representative of current UK practice. Each service followed a different ethos with different practices relating to the transition back to the community. Since the numbers are small, data collected from day patients are presented together and are not separated by site. All hospitals are NHS operated except one which is private and accepts NHS referrals. More details can be found in a previous paper [27].

### Ethics and governance

Ethics approval was granted by the Royal Free Hospital Ethics Committee (08/H0720/41) and site-specific ethics and governance approval was granted on all participating sites. This study was adopted by the Mental Health Research Network (MHRN) and Clinical Studies Officers supported the study.

### Assessment measures

Patients were assessed at admission and after discharge from hospital. Patients completed the first assessment on admission to hospital after providing consent for the study. Patients were not re-contacted by the study team until time of discharge. The hospital notified the treatment team that the patient had been discharged and questionnaires were subsequently sent to the patient at their home.

### Primary outcomes

Body Mass Index (BMI): BMI (weight/height^2^) was obtained from the medical notes for each patient at admission and at discharge. This was available for all patients at admission. It was necessary to use self-reported weight for N = 14 patients (8%) at discharge.

Eating Disorder Examination – Questionnaire (EDEQ): A self-report measure assessing ED symptoms over the previous 28 days [28]. This instrument has good reliability and validity in ED samples [29]. High scores indicate greater ED psychopathology.

Depression, Anxiety and Stress Scale (DASS): A 21-item self-report measure to assess mood state over the past seven days using a 4-point Likert scale [30]. Total score as a measure of general distress or depression, anxiety and stress subscales can be used. High scores indicate higher symptomatology. This measure has good reliability and validity [30,31].

Motivation to change: Patients are asked to rate, on Likert scales measuring 1 (not at all) to 10 (very), their importance and confidence in changing their ED behaviours.

World Health Organization – Quality of Life Questionnaire (brief version) (WHOQOL): Items are rated on a 5-point Likert scale pooled in four domains: physical health, psychological, social relationships and environment. Two single items evaluate the ‘Overall Quality of Life’ and ‘General Health’ facet. Good psychometric data have been reported for this scale [32]. High scores indicate better quality of life.

Levels of Expressed Emotion Scale (LEE): This is a 60-item self-report scale for patients to report on the presence of items representing high or low levels of expressed emotion in their carer using a true/false dichotomy. A total score as well as subscales can be derived [33,34]*.*

Psychological Control Scale (Youth self-report; PCS): This 8-item questionnaire assesses the level of perceived psychological control displayed by carers (reported for mother and father separately) [35].

### Statistical analyses

All statistics were carried out using SPSS version 20.0.

Three participants (2 adult inpatients, 1 adolescent inpatient) were censored due to extremely long hospital admissions (duration stay > 132.5 weeks at time of writing). These patients remained in hospital at the time of data analysis and analyses were conducted without their discharge data.

#### Sample description and treatment outcomes

Data were split according to service characteristics (adult vs. adolescent, inpatient vs. day patient) to provide a comprehensive description of the sample characteristics. Paired t-tests and McNemar tests were used to examine change over the treatment period within each group. Direct comparisons of change in symptomatology between adult inpatients, adolescent inpatients, and day patients were not made because of the large variation in sample size. Instead, effect sizes are presented for a descriptive comparison of the size of change between groups. Hochberg’s improved Bonferroni correction was used to adjust for multiple testing. Cohen’s *d* effect sizes are presented for continuous data to enable comparison across sites. Effect sizes are computed using output from paired t-tests and therefore do not consider non-responders.

#### Predictors of outcome

Data on one or more key variables were missing from N=71 patients and these were excluded from the modeling of predictors which therefore included 107 participants.

The data were explored for fit with the assumptions required for a multivariate analysis. Appropriate transformations were applied to produce variables which were normally distributed. Descriptive statistics (mean, standard deviations, skewness and kurtosis) and Pearson correlation analyses between variables were examined. To examine the degree to which individual and interpersonal variables are linked to ED symptoms at discharge, a two-step sequence of hierarchical regression analyses with the EDEQ at discharge as the criterion variable were conducted. All individual variables (BMI on admission, ED symptoms on admission, patient’s distress and confidence to change) were entered in the first block. All interpersonal variables (LEE, social quality of life and perceived psychological control) were entered in the second block.

## Results

### Engagement into the study procedure

**Figure 1:**
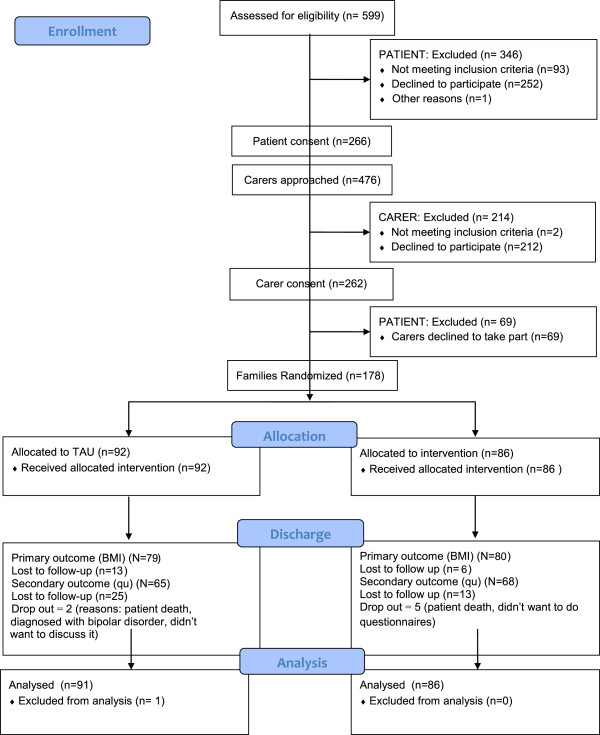
Consort flow diagram.

The consort diagram of the patient and carer flow into the initial phase of the study is shown in Figure 1. In total, 30% of patients who were approached took part in the study. Reasons for not consenting into the study varied but the most common reasons were that patients were discharged prior to consent, didn’t have time to take part in research, or lacked a carer. Also, patients were excluded because their carers did not consent to take part in the study.

### Clinical and demographic data

#### Clinical characteristics of patients at admission

Data are presented for inpatients and day patients (adults) and adolescents (inpatients) separately. The baseline demographic descriptions for the patients are shown in Table 1. Baseline and discharge clinical data are presented in Table 2. Please refer to Additional file 1 for admission characteristics of inpatients, presented by site. Table 3 depicts discharge values and effect sizes for inpatients only by site. There were no significant differences at baseline between those for whom discharge data was available for those for whom data was unavailable.

**Table 1:** Demographic characteristics of patients

	**Adult Inpatients (N=150)**	**Adult Day patients (N=16)**	**Adolescent Inpatients (N=11)**
	Frequency (%)		
Female : Male	95 : 5	100 : 0	91 : 10
Ethnicity			
White (British, Irish, Other)	94	69	100
Asian/Asian British/Mixed	6	31	0
Marital Status			
Married/Living together/In a relationship	22	19	0
Single	75	75	100
Divorced/Separated/Widowed	3	6	0
Highest Qualifications			
None	4	0	30
O-Level/GCSE/A-Level/GNVQ	52	38	60
Diploma/BTEC	10	6	0
University degree or higher	33	56	0
Other	1	0	10
Employment Status			
Paid employment (full-time : part-time)	11 : 5	0 : 13	0 : 0
Homemaker/Unemployed/Sick/Retired	56	62	10
Student	27	25	90
Other	1	0	0
Living Situation			
Living alone	14	19	0
Living with partner	19	12	0
Living with relatives	54	44	89
Other (e.g. hostel, flat with friends)	13	25	11

**Table 2:** **Patient baseline and discharge clinical characteristics: inpatients split by *adult *** **and *adolescent *** site *

	**INPATIENT: ADULT**				**INPATIENT: ADOLESCENT**				**DAYPATIENT: ADULT**			
	**Mean (SD) Admission N =150**	**Mean (SD) Discharge (N = 137)**	**Test statistics**	**ES**	**Mean (SD) Admission N = 11**	**Mean (SD) Discharge (N = 7)**	**Test statistics**	**ES**	**Mean (SD) Admission (N = 16)**	**Mean (SD) Discharge (N = 15)**	**Test statistics**	**ES**
AGE	26.5 (8.9)				15.2 (1.6)				24.7 *(5.3)*			
AGE ONSET	16.8 (5.6)				13.5 (1.4)				16.1 *(4.1)*			
LENGTH ILL (YEARS)	8.2 (8.3)				2.0 (1.7)				7.3 *(7.0)*			
LOWEST BMI	12.6 (1.7)				14.0 (2.0)				14.6 *(1.6)*			
# PREVIOUS ADMISSIONS ^a^	1.0 (0-2.0)				0.4 (0.5)				0 (0-2.0)			
*Treatment*												
LoS (weeks)		26.4 (17.9)				29.0 (11.9)				17.8 *(10.4)*		
*Weight gain*												
BMI	14.0 (1.7)	17.3 (2.1)	**-21.4,** ***p*** **< .001**	**1.7**	15.0 (1.6)	18.5 (1.3)	**-5.6,** ***p*** **= .001**	**3.1**	17.3 *(2.4)*	18.5 *(2.2)*	-2.1, *p* = .059	0.4
Weight (kgs)	38.3 (6.0)	47.1 (7.5)	**-20.6,** ***p*** **< .001**	**1.3**	40.8 (6.1)	50.9 (5.3)	**-5.6,** ***p*** **= .003**	**2.1**	45.1 (7.4)	48.4 (6.0)	-2.0, *p* = .065	0.4
Weekly gain (kg)		0.4 (0.3)				0.5 (0.4)				0.1 (0.3)		
BMI 6 weeks*		15.1 (1.8)				15.1 (1.1)				17.5 *(2.4)*		
6wk rate of weekly weight *		0.6 (0.5)				0.2 (0.2)				-0.2 (0.4)		
*Eating psychopathology (0-6) and behaviours*												
EDE-Q Total	4.2 (1.3)	3.3 (1.6)	**6.5,** ***p*** **< .001**	**-0.6**	4.1 (1.4)	3.5 (1.0)	0.3, *p* = .778	-0.2	4.4 *(1.0)*	3.7 *(1.3)*	**2.7,** ***p*** **= .020**	**-0.7**
EDE-Q Restraint	3.7 (1.8)	2.9 (1.9)	**3.7,** ***p*** **< .001**	**-0.4**	3.7 (2.2)	2.7 (1.2)	0.3, *p* = .802	-0.2	3.6 *(2.0)*	3.3 *(1.4)*	1.4, *p* = .186	-0.2
EDE-Q Eating Concern	3.7 (1.3)	2.8 (1.5)	**6.3,** ***p*** **< .001**	**-0.6**	3.6 (1.5)	3.0 (1.5)	-0.1, *p* = .898	-0.1	4.0 *(1.1)*	3.3 *(1.4)*	**2.3,** ***p*** **= .041**	**-0.7**
EDE-Q Shape Concern	5.0 (1.2)	4.1 (1.8)	**5.9,** ***p*** **< .001**	**-0.5**	5.2 (0.8)	4.8 (0.8)	0.8, *p* = .499	-0.3	5.3 *(0.9)*	4.5 *(1.5)*	**2.8,** ***p*** **= .016**	**-0.8**
EDE-Q Weight Concern	4.5 (1.4)	3.3 (1.7)	**7.2,** ***p*** **< .001**	**-0.7**	4.1 (1.7)	3.7 (1.1)	0.4, *p* = .729	-0.3	4.7 *(1.1)*	3.7 *(1.4)*	**2.9,** ***p*** **= .012**	**-0.8**
OBE: Yes	55 (37%)	37 (31%)	*p* = .440	-0.2	3 (38%)	3 (50%)	*p* = 1.0	0.3	7 *(44%)*	10 *(67%)*	*p* = .250	0.6
LOC: Yes	82 (59%)	62 (56%)	*p* = .871	-0.1	3 (43%)	2 (40%)	*p* = 1.0	-0.1	12 *(75%)*	8 *(52%)*	*p* = .625	-0.6
SIV: Yes	42 (30%)	26 (23%)	*p* = 1.0	-0.2	2 (29%)	1 (20%)	*p* = 1.0	-0.3	6 *(38%)*	5 *(39%)*	*p* = 1.0	0.0
LAX: Yes	29 (20%)	12 (11%)	*p* = .152	-0.4	0	1 (20%)	*p* = 1.0	-	3 *(19%)*	2 *(15%)*	*p* = 1.0	-0.2
DIUR: Yes	8 (6%)	0	*p* = .063	-	0	0	-	-	1 *(6%)*	2 *(15%)*	*p* = 1.0	-0.5
EXC. EX: Yes	83 (58%)	41 (37%)	***p*** **= .012**	**-0.5**	5 (63%)	1 (20%)	*p* = .500	-1.2	7 *(44%)*	7 *(54%)*	*p* = 1.0	0.3
*Mood and Quality of life*												
DASS total	77.8 (27.9)	64.8 (31.7)	**4.9,** ***p*** **< .001**	**-0.4**	75.3 (33.4)	36.0 (16.4)	1.5, *p* = .233	-0.9	79.1 *(25.2)*	67.5 *(33.0)*	1.1, *p* = .277	-0.4
DASS Depression	28.4 (11.3)	24.3 (12.7)	**3.8,** ***p*** **< .001**	**-0.3**	30.5 (11.8)	14.0 (8.9)	1.2, *p* = .309	-0.9	29.5 *(10.8)*	25.1 *(14.7)*	1.0, *p* = .316	-0.4
DASS Anxiety	20.3 (10.6)	15.7 (11.7)	**4.5,** ***p*** **< .001**	**-0.4**	15.5 (12.4)	8.4 (5.2)	*p* = .285	-0.4	20.1 *(7.6)*	18.9 *(10.1)*	-0.4, *p* = .726	-0.3
DASS Stress	29.1 (9.7)	24.7 (10.7)	**4.3,** ***p*** **< .001**	**-0.4**	29.3 (10.9)	13.6 (6.5)	1.9, *p* = .155	-1.3	29.5 *(8.6)*	23.5 *(9.7)*	1.7, *p* = .108	-0.7
WHO QoL (1-5)	2.4 (1.0)	3.2 (0.9)	**-6.7,** ***p*** **< .001**	**0.8**	2.3 (0.9)	4.0 (1.0)	-2.0, *p* = .141	1.6	2.9 *(1.0)*	2.8 (1.3)	0.4, *p* = .721	-0.1
WHO HEALTH (1-5)	2.3 (0.9)	2.9 (1.0)	**-5.0,** ***p*** **< .001**	**0.5**	3.0 (1.2)	4.2 (0.8)	-1.2, *p* = .308	0.6	2.4 *(1.0)*	2.3 (1.0)	0.4, *p* = .721	-0.1
WHO Psychological	26.2 (17.5)	35.5 (19.5)	**-5.6,** ***p*** **< .001**	**0.5**	26.0 (18.2)	46.7 (20.5)	-0.7, *p* = .519	0.5	22.1 *(17.3)*	26.3 (20.7)	-0.9, *p* = .378	0.2
WHO Social	40.2 (22.1)	40.8 (21.7)	0.6, *p* = .581	0.1	38.0 (31.6)	41.7 (25.7)	0.7, *p* = .537	0.3	33.3 *(18.0)*	26.3 (17.3)	0.3, *p* = .764	-0.1
WHO Physical	51.0 (20.0)	62.2 (20.8)	**-5.3,** ***p*** **< .001**	**0.5**	67.0 (20.4)	86.4 (8.5)	-1.6, *p* = .207	0.3	46.2 *(18.1)*	45.3 (22.6)	0.7, *p* = .484	-0.1
WHO Environment	52.7 (16.9)	61.2 (17.0)	**-5.2,** ***p*** **< .001**	**0.4**	56.6 (14.0)	72.5 (19.8)	-1.6, *p* = .215	0.7	53.6 *(9.9)*-0.2	50.3 (16.1)	1.1, *p* = .296	
*Motivation (1-10)*												
Importance to change	8.0 (2.3)	7.5 (2.5)	**2.6,** ***p*** **= .010**	**-0.3**	6.3 (4.3)	4.2 (3.3)	2.1, *p* = .127	-0.6	8.9 *(1.1)*	8.4 *(2.5)*	0.5, *p* = .603	-0.2
Confidence to change	5.5 (2.6)	5.4 (2.5)	1.2, *p* = .222	-0.1	5.0 (2.5)	6.2 (2.3)	-0.9, *p* = .448	0.6	5.0 *(2.0)*	4.7 *(2.6)*	0.4, *p* = .711	-0.3
*Perceived support from carers*												
LEE primary carer	20.0 (15.4)	18.3 (13.8)	1.3, *p* = .182	-0.1	20.6 (15.0)	14.7 (6.7)	0.2, *p* = .858	-0.1	19.0 (9.9)	22.5 (12.9)	-1.2*,* p = .249	0.3
PCS Mother	2.4 (1.0)	2.4 (1.0)	-0.5, *p* = .633	0.0	2.0 (0.8)	1.8 (0.1)	0.5, *p* = .670	-0.04	2.5 (0.9)	2.5 (0.9)	-0.1, *p* = .902	-0.02
PCS Father	2.2 (1.0)	2.1 (0.9)	0.5, *p* = .618	-0.04	1.9 (0.5)	1.9 (0.3)	-0.04, *p* = .973	0.03	2.4 (1.0)	2.5 (1.3)	-0.4, *p* = .708	-0.1

* Not every participant answered every question; ^a^ Median and IQR reported. ^b^ data for one site not presented in isolation as only 2 participants were inpatients; day patients are not included in individual site calculations; ES: Effect size; SD: Standard Deviation; N = sample number included; BMI: Body Mass Index; EDE-Q Eating Disorder Examination Questionnaire; OBE: Objective binge episodes with loss of control; LOC: Loss of control other than binge; SIV: Self-induced vomiting; LAX: Laxative use; EXER: Excessive exercise; DASS: Depression, Anxiety and Stress Scale; WHO: World Health Organization; QoL: Overall quality of life (1–5 high); HEALTH: Overall satisfaction with health (1–5 high); LEE: Levels of Expressed Emotion; PCS: Psychological Control Scale; Analyses for change over treatment period for adolescent and day patient samples were conducted using t-tests (data presented in table) and Wilcoxin Signed Ranks test for non-parametric data because of small sample sizes. There were no differences in the pattern of results. Bold values indicate a statistically significant result.

**Table 3:** **Descriptive data (mean, standard deviation) at discharge and effect sizes for change pre- and post- inpatient treatment by centre: *inpatients only***^a^

	**1**	**2**	**3**	**4**	**5**	**6**	**7**	**8**	**9**	**10**	**11**	**12**	13*	14*
	**N = 21**	**N = 17**	**N = 16**	**N = 16**	**N = 12**	**N = 15**	**N = 11**	**N = 11**	**N = 7**	**N = 6**	**N = 3**	**N = 2**	**N = 5**	**N = 2**
Age at admission	27.9 (8.2)	33.1 (14.3)	24.3 (8.2)	24.6 (9.6)	23.7 (5.0)	23.4 (5.3)	23.4 (7.4)	30.8 (6.7)	30.5 (8.0)	21.2 (3.3)	25.0 (9.6)	24.5 (7.8)	15.6 (1.5)	15.3 (0.6)
Admission (weeks)	38.1 (25.4)	19.9 (16.5)	35.2 (13.2)	24.7 (16.3)	21.9 (20.4)	27.7 (14.3)	22.1 (10.1)	16.6 (9.2)	22.1 (8.8)	21.3 (13.7)	22.4 (9.5)	15.8 (3.7)	30.0 (13.7)	26.5 (8.2)
BMI	17.4 (2.3)	17.7 (2.2)	17.2 (1.9)	17.4 (1.6)	16.9 (1.1)	17.5 (2.1)	18.0 (3.7)	15.6 (1.6)	16.3 (1.5)	18.4 (1.6)	17.9 (1.3)	16.2 (0.7)	18.6 (1.2)	18.4 (2.0)
*d*	*1.4*	*1.6*	*2.4*	*2.5*	*2.4*	*1.8*	*0.8*	*1.8*	*1.6*	*2.9*	*2.4*	*-*	*3.8*	*-*
Weight (kgs)	46.3 (6.7)	47.3 (6.0)	47.4 (6.1)	47.1 (5.6)	47.6 (5.1)	44.7 (6.1)	53.5 (12.1)	42.6 (4.3)	43.9 (6.4)	51.7 (6.9)	45.6 (4.2))	44.8 (2.0)	52.3 (5.3)	49.7 (6.2)
Weekly gain (kgs)	0.3 (0.2)	0.6 (0.4)	0.3 (0.1)	0.4 (0.2)	0.5 (0.2)	0.4 (0.3)	0.5 (0.2)	0.6 (0.2)	0.3 (0.2)	0.8 (0.5)	0.4 (0.1)	0.3 (0.1)	0.6 (0.4)	0.2 (0.1)
EDE-Q Total	3.5 (1.4)	3.2 (1.9)	3.8 (1.0)	2.5 (1.9)	3.4 (1.4)	3.1 (1.7)	3.6 (1.5)	2.7 (1.1)	4.4 (1.1)	0.8 (0.3)	3.8 (0.8)	5.2 (0.8)	3.3 (1.0)	4.6
*d*	*-0.5*	*-0.3*	*-0.5*	*-0.9*	*-1.2*	*-0.3*	*-0.3*	*-1.1*	*-0.5*	*-1.5*	*-0.9*	*-*	*-0.5*	*-*
OBE N (%)	8 (40%)	4 (29%)	4 (31%)	3 (30%)	3 (25%)	3 (23%)	3 (25%)	5 (50%)	1 (20%)	1 (20%)	1 (50%)	0	2 (50%)	1
SBE N (%)	15 (71%)	5 (42%)	8 (73%)	3 (30%)	7 (64%)	3 (30%)	8 (67%)	3 (38%)	4 (100%)	1 (20%)	1 (25%)	2 (100%)	1 (25%)	1
SIV N (%)	5 (24%)	1 (8%)	2 (18%)	3 (30%)	3 (27%)	0	6 (50%)	1(12.5%)	2 (50%)	1 (20%)	2 (50%)	0	1 (25%)	0
LAX N (%)	3 (14%)	2 (17%)	2 (18%)	1 (10%)	1 (9%)	0	1 (8%)	0	2 (50%)	0	0	0	1 (25%)	0
EXERCISE N (%)	7 (33%)	4 (33%)	6 (55%)	4 (40%)	4 (40%)	3 (30%)	6 (46%)	3 (38%)	3 (75%)	0	0	1 (50%)	0	1
														
DASS total	74.1 (28.6)	54.7 (28.5)	77.8 (23.9)	58.0 (31.7)	68.0 (25.3)	66.2 (37.1)	62.3 (28.9)	54.0 (34.6)	88.0 (41.9)	12.8 (6.6)	63.0 (19.9)	109.0 (9.9)	39.5 (16.6)	22.0
*d*	*-0.2*	*-0.3*	*-0.2*	*-0.5*	*-0.7*	*-0.2*	*-0.7*	*-0.9*	*0.0*	*-0.7*	*-0.9*	*-*	*-0.9*	*-*
WHO QoL	3.0 (0.8)	3.8 (0.5)	2.9 (0.7)	3.7 (0.7)	3.3 (1.0)	3.1 (1.0)	2.9 (1.1)	3.2 (1.3)	2.8 (0.8)	4.6 (0.5)	3.3 (0.5)	1.5 (0.7)	3.8 (1.0)	5.0
*d*	*0.7*	*0.8*	*0.8*	*1.2*	*1.3*	*0.7*	*0.3*	*1.3*	*-0.3*	*1.4*	*0*	*-*	*1.2*	*-*
WHO Health	2.6 (0.9)	3.6 (0.9)	2.5 (1.0)	3.3 (0.7)	2.6 (0.9)	2.8 (1.2)	3.2 (0.7)	3.2 (1.0)	2.6 (0.5)	4.0 (0)	2.5 (1.0)	1.5 (0.7)	4.3 (1.0)	4.0
*d*	*0.6*	*0.9*	*0.4*	*0.5*	*0.8*	*0.3*	*0.1*	*1.8*	*0.7*	*-*	*0.3*	*-*	*0.2*	*-*
WHO Psychological	33.3 (15.8)	40.3 (15.6)	25.0 (13.2)	45.3 (16.2)	32.2 (16.0)	30.0 (22.8)	32.6 (17.1)	41.8 (24.6)	27.5 (18.3)	75.0 (12.8)	34.4 (6.3)	4.2 (0)	40.6 (17.8)	70.8
*d*	*0.5*	*0.6*	*0.2*	*1.0*	*0.4*	*0.2*	*0.4*	*0.7*	*0.4*	*1.0*	*1.6*	*-*	*0.2*	*-*
WHO Social	36.9 (25.6)	43.4 (22.6)	37.9 (17.2)	44.2 (23.6)	41.3 (10.9)	36.3 (16.7)	41.3 (20.0)	48.3 (28.5)	29.2 (16.0)	65.0 (34.6)	43.8 (14.2)	29.2 (29.5)	39.6 (29.2)	50.0
*d*	*0.1*	*0.4*	*0.2*	*0*	*-0.1*	*0.4*	*0.1*	*0.3*	*-0.1*	*0.6*	*0.1*	*-*	*-0.7*	*-*
WHO Physical	55.7 (23.4)	72.9 (13.3)	58.1 (13.7)	72.2 (13.1)	59.1 (22.6)	61.9 (25.8)	62.2 (14.5)	63.1 (25.6)	51.4 (21.8)	88.6 (13.7)	58.9 (16.1)	25.0 (20.2)	87.5 (9.4)	82.1
*d*	*0.4*	*1.2*	*0.1*	*0.7*	*0.4*	*0.6*	*0.2*	*0*	*0.6*	*0.5*	*0.4*	*-*	*0.0*	*-*
WHO Environment	55.8 (16.7)	71.6 (9.6)	54.0 (9.8)	58.9 (16.1)	67.9 (16.5)	61.6 (15.5)	62.2 (16.4)	62.5 (20.0)	51.3 (19.3)	72.5 (23.7)	70.1 (21.0)	35.9 (24.3)	75.8 (21.3)	59.4
*d*	*0.3*	*1.0*	*0.3*	*0.4*	*0.6*	*0.4*	*0.2*	*0.6*	*0.3*	*0.3*	*0.5*	*-*	*0.6*	*-*
Importance to change	7.9 (2.6)	7.8 (2.6)	7.0 (2.3)	6.9 (3.0)	8.0 (1.3)	8.2 (2.0)	6.6 (3.5)	8.0 (1.7)	6.8 (0.5)	6.2 (3.8)	7.0 (2.6)	10.0 (0)	4.0 (3.8)	5.0
*d*	*-0.3*	*-0.3*	*-0.6*	*-0.2*	*-0.1*	*-0.6*	*-0.2*	*-0.3*	*-*	*0*	*-0.1*	*-*	*-0.3*	*-*
Confidence to change	5.4 (2.3)	4.8 (2.6)	4.2 (1.9)	6.2 (1.2)	6.2 (2.3)	5.5 (3.1)	4.1 (2.9)	6.8 (2.0)	4.0 (1.8)	8.8 (1.3)	4.8 (2.4)	6.0 (1.4)	5.3 (1.0)	10.0
*d*	*0.2*	*-0.3*	*-0.5*	*0.0*	*0.2*	*0.2*	*-0.4*	*0.1*	*0.5*	*0.5*	*-0.1*	*-*	*0*	*-*
LEE Carer 1	19.4 (14.5)	14.2 (10.9)	25.7 (11.2)	17.9 (16.6)	16.9 (12.5)	19.3 (16.6)	22.8 (14.7)	11.7 (11.8)	17.4 (15.5)	7.2 (7.2)	15.8 (10.9)	31.5 (20.5)	15.6 (7.4)	11.0
*d*	*0.4*	*0.1*	*0.1*	*0.3*	*0.4*	*-0.1*	*0.1*	*-0.4*	*0.0*	*-0.8*	*0.3*	*-*	*-0.4*	*-*
PCS mother	2.6 (1.0)	2.0 (1.0)	2.6 (0.8)	2.5 (1.3)	2.1 (1.0)	2.6 (1.2)	2.5 (0.8)	1.9 (0.8)	1.6 (0.8)	2.5 (0.7)	1.9 (0.8)	4.3 (0.9)	1.9 (0.4)	2.0
*d*	*0.1*	*0.1*	*0.0*	*0.2*	*0.3*	*0.0*	*0*	*0.0*	*0.0*	*0.5*	*0.5*	*-*	*0*	*-*
PCS father	2.3 (0.7)	1.9 (0.9)	2.7 (1.0)	2.2 (1.4)	1.8 (0.5)	1.9 (0.9)	2.0 (0.7)	1.8 (1.0)	1.8 (1.3)	2.2 (1.3)	1.9 (0.3)	2.4 (0.5)	2.1 (0.1)	1.4
*d*	*0.1*	*0.1*	*0.2*	*-0.1*	*0.2*	*0.1*	*0.1*	*0.1*	*0.0*	*0*	*0.3*	*-*	*0.6*	*-*

^a^ Not every participant answered every question; * Adolescent centre; N = sample number included; d = Cohen’s d effect sizes for paired t-tests. Effect sizes are therefore completed for complete admission-discharge pairs. BMI: Body Mass Index; EDE-Q Eating Disorder Examination Questionnaire; OBE: Objective binge episodes with loss of control; LOC: eating with loss of control other than binge; SIV: Self induced vomiting; LAX: Laxative use; EXER: Excessive exercise; Note: diuretic use was not endorsed by anyone. DASS: Depression, Anxiety and Stress Scale; WHO: World Health Organisation; QoL: Overall quality of life (1-5 high); HEALTH: Overall satisfaction with health (1-5 high); LEE: Levels of Expressed Emotion; PCS: Psychological Control Scale.

#### The case mix of adult inpatients

Overall, inpatients in adult units (aged 16–62 years) are admitted to hospital with very low BMI. Seventy-nine percent of patients had a BMI ≤ 15. Five patients (3%) had a BMI above 17.5 and had been admitted to hospital due to significant and rapid weight loss or extreme binge-purge symptoms. Both the specific ED and more general aspects of psychopathology were spread across the total range of the scales but the large majority of patients scored in the severe to extremely severe ranges. Patients scored moderately high on “importance to change” but lower on “confidence to change”.

The case mix at admission varied somewhat between services. BMI on admission was slightly lower at 2 sites in particular (7, 9) and participants were slightly older in 3 centres (2, 7, 9). This may reflect their roles as national specialist centres. Patients from almost all centres reported high ED psychopathology and objective bingeing, purging and excessive exercise. Mean levels of depression and anxiety were in the severe range for most sites.

##### Adult vs Adolescent

Adolescents (aged 13–17 years old) tended to have a higher BMI at admission (*d* = -0.6) and reported lower ‘importance to change’ than adults.

##### Inpatients vs. day patients

Day patients had higher BMI at admission, higher lowest lifetime BMI and fewer previous admissions than adult inpatients (50% of day patients compared to 70% of inpatients).

##### Interpersonal factors

Perceived expressed emotion (criticism and overprotection and perceived psychological control) was moderately high and similar across groups.

#### Service level information

#### Treatment response

### Adult inpatients

#### Length of admission

There was considerable individual variation in length of admission (4 – 141.6 weeks). There was large variation in the mean length of admissions between sites (15.8 – 38.1 weeks) where some sites reported twice the length of admissions than others. Thirteen patients (9%) received a stepped care treatment package (inpatient for an average of 24.2 weeks followed by 16.2 weeks day patient care).

### BMI

Inpatient treatment was successful for BMI (huge effect size). However, there was a large range in individual discharge BMI (11.9 – 24.4). The average rate of weekly weight gain over the duration of the admission varied widely between individuals (0.2 – 1.7 kg per week.) Effect sizes for change in BMI were large for all sites but there was a two-fold difference in the size of the effect between some sites. The rate of weight gain was lower than the minimum recommendation by NICE (0.5 kgs) in eight centres (seven adult).

The majority of adult inpatients remained in the AN BMI range at discharge from hospital (58%). Twenty (14%) of these had a discharge BMI of ≤ 15 and a further 62 (44%) had a BMI ≤ 17.5. Only 22 percent (N = 31) of patients had a BMI >19 at discharge.

### Eating psychopathology

Scores on EDEQ improved significantly with moderate sized effects. The only ED behaviour for which there was a significant reduction in the number of patients endorsing the behaviour was excessive exercise. There was a two-three fold difference in the size of effect between some centres.

### Mood, quality of life and motivation to change

Mean discharge scores for depression and anxiety remained in the severe range and ‘stress’ scores fell within the upper moderate range. There was no change in social wellbeing. Ratings on ‘importance to change’ significantly reduced over the treatment period and there were no changes in ‘confidence to change’.

There was a three-fold difference in the size of effects for mood and quality of life outcomes across centres. There was an increase in ‘importance to change’ in only two centres and an increase in ‘confidence to change’ in five centres.

#### Family relationship

Overall, there were no changes in perceived expressed emotion or perceived psychological control for any group.

### Adolescent inpatients

#### Length of stay

One adolescent patient remained in hospital at the time of data analysis. There was a tendency for adolescents to have a longer admission than adults but there was wide variation (15.9 – 56.0 weeks).

#### Response to treatment

### BMI

The overall increase in BMI in adolescents was double that of the adult sample. The rate of weekly weight gain was slightly more than adults.

### Eating psychopathology

There were small reductions in ED specific psychopathology and, like adults, the only behavioural measure to show change was excessive exercise.

### Mood, quality of life and motivation to change

Effect sizes for improvements in mood were larger for adolescents than for adults and there was improvement in all WHO Quality of Life subscales. ‘Importance to change’ reduced but, unlike adults, ‘confidence to change’ increased over the treatment period.

### Inpatient vs Day patient

#### Length of stay

The length of stay was shorter for day patients than inpatients (*U* = 786.5, *p* = .026).

#### Response to treatment

Overall, specific and general symptoms improved with day patient care but the effects were smaller than for inpatients.

### BMI

There was a moderate increase in BMI but 40% of day patients remained in the AN weight range at discharge. The weekly rate of weight gain was modest with large individual variation (0.1kg; range: -0.2 – 1.2).

### Eating psychopathology

Change in ED specific psychopathology was significant (large effects) but 40% remained above the clinically significant threshold. There was no change in number of patients engaging in ED behaviours.

### Mood, quality of life and motivation to change

There were small to medium improvements in mood and quality of life. Both ‘importance to change’ and ‘confidence to change’ reduced over the treatment period.

#### Predictors of response to treatment

#### Preliminary analysis

A skewed distribution was found for expressed emotion (LEE). Square root transformations were applied to this score to better approximate a normal distribution for the analyses involving this variable. There was no indication of multicollinearity as tolerance statistics were above .20, and variance inflation factors were below 4. Table 2 shows descriptive statistics (mean and SD) of all study variables.

#### Correlation analysis

Table 4 reports correlations between the study variables. We found that ED symptoms at discharge were positively correlated with ED symptoms on admission, patients’ mood and expressed emotion, and negatively related to patients’ confidence in ability to change.

**Table 4:** Descriptive statistics and correlations among the study variables (n = 107)

	***Mean***	***SD***	***1***	***2***	***3***	***4***	***5***	***6***	***7***	***8***
1. BMI on admission	14.25	2.38	-							
2. ED symptoms admission (EDEQ)	4.16	1.28	.100	-						
3. Distress (DASS)	74.50	28.35	.042	.5542^**^	-					
4. Confidence in ability to change	5.52	2.68	-.098	-.446^**^	-.531^**^	-				
5. Expressed Emotion (LEE)	19.92	16.43	.020	.164	.376^**^	-.254^**^	-			
6. Social Quality of Life (WHO QoL)	10.34	3.40	-.084	-.171	-.317^**^	.310^**^	-.410^**^	-		
7. Mothers' Psychological Control	2.33	1.04	.075	.143	.284^**^	-.130	.635^**^	-.250^**^	-	
8. Fathers' Psychological Control	2.16	.90	.136	.097	.123	-.269^**^	.384^**^	-.174	.455^**^	-
9. ED symptoms at discharge (EDEQ)	3.36	1.44	.096	.536^**^	.575^**^	-.512^**^	.256^**^	-.097	.128	-.008

BMI = Body mass Index in admission; EDEQ: Eating Disorder Examination Questionnaire; DASS: Depression, Anxiety and Stress Scale; LEE: Levels of Expressed Emotion; WHO: World Health Organization; QoL: Quality of Life;

* p < .05; ** p < .01.

#### Regression analysis

Table 5 summarises the results of the hierarchical regression analyses in which four individual factors and four interpersonal factors were entered consecutively to explain ED symptoms at discharge. The cumulative variance accounted for by the entire model, as well as the incremental for each block are presented. Overall, individual variables accounted for a significant amount of variance (42%, p < .001) in predicting ED symptoms at discharge. In this equation, ED symptoms on admission (β = .264, p = < .01), patients distress (β = .311, p = < .01) and confidence in ability to change (β = -.227, p = < .05) made significant contributions to explain ED symptoms at discharge. In the second equation, ED symptoms on admission (β = .264, p = < .01), patients distress (β = .285, p = < .01) and confidence in the ability to change (β = -.295, p = < .01) remained as predictors of ED symptoms at discharge, and the subsequent entry of interpersonal variables accounted for significant incremental variance over and above individual factors in predicting 6% (p < .05) additional variance. This can be largely attributed to the significant contribution of expressed emotion (β = .205, p <.05), social quality of life (β = .178, p < .05), and perceived psychological control in fathers (β = -.189, p < .05).

**Table 5:** Regression analysis on eating psychopathology at discharge

	***B***	***SE***	***β***	***t***	***p***	***Adj R***^***2***^	***F***	***p***	
*DV: EDEQ at discharge*									
Step 1							.417	19.983	.000
	BMI admission	.021	.045	.034	.455	.650			
	Eating disorder symptoms in admission	.297	.102	.264	2.904	.005			
	Distress	.016	.005	.311	3.246	.002			
	Confidence in ability to change	-.122	.048	-.227	-2.518	.013			
Step 2							.456	12.114	.000
	BMI admission	.041	.044	.067	.924	.358			
	Eating disorder symptoms in admission	.297	.099	.264	2.997	.003			
	Distress	.014	.005	.285	2.884	.005			
	Confidence in ability to change	-.159	.049	-.295	-3.216	.002			
	Expressed Emotion	.171	.085	.205	2.021	.046			
	Social Quality of Life	.076	.035	.178	2.193	.031			
	Mothers’ Psychological Control	-.046	.137	-.033	-.334	.739			
	Fathers’ Psychological Control	-.303	.136	-.189	-2.231	.028			

## Discussion

This study represents a unique collaboration of major UK ED treatment centres and describes a cross-section of severely ill patients with AN at admission and discharge from specialist hospital treatment.

### Patient characteristics

The majority of patients had a severe and enduring form of illness and had failed to respond to earlier intensive treatment. They had high levels of functional impairment and most were living with their family of origin prior to their admission. Day patients had a higher weight at admission and less severe historical illness characteristics (lowest BMI, previous admissions) than inpatients. Adolescents had a higher BMI and less protracted illness than adults.

### Change in symptoms at discharge - inpatients

The change in clinical symptoms during the period of treatment was large for weight gain but modest for most other symptoms. The majority of patients were discharged when their BMI remained within the AN diagnostic weight range (≤ 17.5) and two thirds of the sample remained within the clinical range of general and specific psychopathology. Overall, the proportion of people engaging in compensatory behaviours did not show great change at discharge. Perceptions of quality of life and health improved but all domains remained low upon discharge relative to population norms [36]. However, this might be expected since patients need to reconnect socially and/or professionally having recently been discharged. Similarly, since psychotherapy sometimes needs a certain physical health to work in depth on psychological symptoms, it is possible that the lack of improvement in ED and mood symptoms is associated with the discharge weight of the group remaining within the pathological ED criteria. Patients’ weight and symptom status at discharge is of concern given previous reports that low weight predicts relapse [23,37,38].

### Change in symptoms at discharge - service level day patients/adolescents

BMI increase was smaller in day patients than in inpatients and at end of treatment, 40% of day patients remained within an anorexic BMI range. Day patients remained symptomatic at discharge and quality of life was poor. The change in BMI in the adolescent inpatient group was twice that attained for adults; this was associated with an elevated rate of weight gain and a small increase in the length of stay. There was also a larger improvement in mood and quality of life, and ‘confidence to change’ also increased over the treatment period which was not seen in the adults.

### Predictors of ED symptoms at discharge

Clinical and social parameters predicted symptom change and some of these have the potential to be modified by change in clinical policy or practice. The level of patient’s mood psychopathology, confidence, social quality of life, perceived level of father’s control and carer expressed emotion predicted ED symptoms at discharge. The level of confidence has previously been found to predict the response to inpatient care [39] and has been found to have implications for post-inpatient outcomes [23]. Social function and family response to the illness (expressed emotion, father’s psychological control) predicted outcome and this supports the interpersonal maintenance model of EDs [24,25]. Carer expressed emotion has previously been found to contribute to the outcome of adolescent AN [40-42] and adults with bulimia nervosa (BN) admitted for inpatient care [43]. It is possible that if inpatient treatment targeted these psychosocial factors, and were inclusive of families, outcomes would be improved.

### Clinical implications

Inpatient care is able to produce an improvement in physical health but psychological and social health remains impaired. There are several ways in which treatment could be focused on those factors that might modify the response to inpatient care. Strategies to improve confidence to change such as motivational interviewing may be of value to improve outcome [44-46]. Targeting interpersonal relationships with interventions that reduce expressed emotion [47,48] may be of benefit. Strategies included in the recovery approach may reduce the social isolation [24,49] and loneliness [50] that contribute to the poor social quality of life.

### Limitations

There are limitations of this study. Primarily, the data do not represent all patients admitted but only the group who consented to be involved in a study that involves their carers. It is possible that the sample is therefore biased towards including carers who were more actively involved in their loved one’s care. The samples recruited from each site or type of service were small, limiting the power to examine service level predictors. The percentage of adolescent inpatients and day patients is also small in comparison to the adult inpatient sample size which limits the overall outcome interpretation. All psychological outcomes were measured using self report questionnaires. Clinical interviews would improve reliability of findings. There were missing data for some of the predictor variables of interest, reducing data available for the regression analysis. However, it is a strength of the study that discharge data were collected from patients regardless of whether they completed the full inpatient/day patient programme, so it is representative of those who leave hospital treatment for AN. Data on whether patients took their own discharge, whether clinicians suggested an early discharge or whether they completed the programme were not assessed in this study since the information was not available for all sites and the definitions of each of these premature terminations of treatment are variable. It would be of interest to examine whether outcomes differed between these groups in future studies. Similarly, it would be interesting to examine outcomes based on different therapeutic approaches within and between sites. Lastly, we did not differentiate between patients who were admitted on a voluntary basis and those detained under the Mental Health Act. Despite these limitations, these data are a realistic reflection of current NHS practice of inpatient care in the UK and this paper presents a platform for future research in the area.

## Conclusion

Overall, the response to inpatient treatment was modest particularly in adults with a severe enduring form of illness. Adolescents had a better response. Although inpatient treatment produces an improvement in physical health, there was less improvement in other ED and mood symptoms. As predicted by the interpersonal maintenance model, carer behaviour may influence the response to inpatient care, as may improved social functioning and confidence to change. Interventions targeting these factors might improve the treatment response.

## Competing interests

The authors declare that they have no competing interests.

## Author’s contributions

EG was a project co-ordinator for the study and was involved in data collection, data entry and data analysis. She was involved in the interpretation of data and preparation of draft and final manuscript for submission. RH is a project co-ordinator for the study and was involved in data collection and data entry. She was involved in the interpretation of data and preparation of draft and final manuscript for submission. SR was a project co-ordinator for the study and was involved in data collection and data entry. She also provided critical appraisal on draft manuscripts and approval for final submission. LS was involved in data analysis and interpretation, contributed to the preparation of draft manuscript and provided approval for final submission. JA, NB, FC, KG, JM, KM, DR, SS, CS, SS, LW are the principal investigators for the services from which we recruited. They all oversaw and were actively involved in data collection, liaising with project co-ordinators and Mental Health Research Network clinical officers and all of them provided critical appraisal on draft manuscripts and approval for final submission. JA also contributed to the interpretation of the data. US was involved in the study conception and design, interpretation of data and in the critical appraisal of drafts and approval for final submission. JT oversaw the project and was involved in study conception, design, and supervision of project progress. She was involved in the interpretation of data, draft preparation, critical appraisal of content and approval of final draft for submission. She was also principal investigator at Bethlem Royal Hospital, South London and Maudsley NHS Trust. All authors read and approved the final manuscript.

## Pre-publication history

The pre-publication history for this paper can be accessed here:

http://www.biomedcentral.com/1471-244X/13/287/prepub

## Supplementary Material

Addittional file 1: **Patient baseline clinical characteristics** ***: inpatients only*** **by participating centre ^a^ b.** Click here for file
