# Supplementary material for: A multi-centre cohort study of short term outcomes of hospital treatment for anorexia nervosa in the UK
Source: BMC Psychiatry. 2013 Nov 7;13:287. doi: 10.1186/1471-244X-13-287 (PMC3871017; doi:10.1186/1471-244X-13-287)
Supplement: Addittional file 1: — Patient baseline clinical characteristics : inpatients only by participating centre a b. [file 1471-244X-13-287-S1.docx]

Additional file 1. Patient baseline clinical characteristics*: Inpatients only* by participating centre ª (Mean, standard deviation)

|  | 1  N = 26 | 2  N = 17 | 3  N = 16 | 4  N = 16 | 5  N = 15 | 6  N = 15 | 7  N = 13 | 8  N = 11 | 9  N = 8 | 10  N = 6 | 11  N = 5 | 12  N = 2 | 13*  N = 7 | 14*  N = 4 |
| --- | --- | --- | --- | --- | --- | --- | --- | --- | --- | --- | --- | --- | --- | --- |
| BMI | 14.1 *(1.8)* | 14.7 *(1.7)* | 13.1 *(1.1)* | 13.8 *(1.2)* | 14.3 *(1.6)* | 14.1 *(1.6)* | 14.6 *(2.5)* | 12.7 *(1.7)* | 13.5 *(1.8)* | 14.0 *(1.0)* | 14.9 *(1.6)* | 14.3 *(0.7)* | 14.2 *(1.1)* | 16.2 *(1.4)* |
| Weight | 38.8 *(7.4)* | 39.1 *(4.3)* | 35.4 *(5.5)* | 37.3 (5.2) | 40.5 *(5.6)* | 37.9 *(4.2)* | 41.5 *(7.5)* | 34.9 *(5.6)* | 36.0 *(6.8)* | 39.2 *(5.0)* | 38.3 *(4.8)* | 39.5 *(2.1)* | 38.5 *(6.6)* | 43.1 *(5.4)* |
| Age | 27.9 *(8.2)* | 33.1 *(14.3)* | 24.3 *(8.2)* | 24.6 *(9.6)* | 23.7 *(5.0)* | 23.4 *(5.3)* | 23.4 *(7.4)* | 30.8 *(6.7)* | 30.5 *(8.0)* | 21.2 *(3.3)* | 25.0 *(9.6)* | 24.5 *(7.8)* | 15.6 *(1.5)* | 15.3 *(0.6)* |
| Age of onset | 17.7 *(5.3)* | 18.7 *(7.1)* | 16.3 *(2.2)* | 15.7 *(3.9)* | 17.8 *(5.9)* | 14.8 *(2.8)* | 18.2 *(6.1)* | 14.9 *(2.8)* | 18.4 *(12.0)* | 17.0 *(5.4)* | 15.8 *(4.1)* | 8.0 *(4.2)* | 14.0 *(1.3)* | 12.8 *(1.3)* |
| Length of illness (years) | 8.1 *(6.5)* | 12.5 *(12.8)* | 5.5 *(4.3)* | 7.2 *(10.5)* | 4.2 *(3.1)* | 8.9 *(6.0)* | 6.5 *(5.2)* | 13.5 *(10.0)* | 12.2 *(9.5)* | 4.3 *(4.8)* | 5.0 *(5.6)* | 5.0 *(4.2)* | 1.8 *(1.6)* | 2.3 *(2.0)* |
| Lowest lifetime BMI | 12.9 *(1.9)* | 13.1 *(1.3)* | 12.3 *(1.4)* | 12.9 *(1.9)* | 13.3 *(1.1)* | 12.1 *(1.6)* | 13.1 *(1.5)* | 11.7 *(2.1)* | 11.8 *(1.8)* | 12.6 *(2.0)* | 13.4 *(1.3)* | 10.4 *(0.1)* | 13.0 *(0.9)* | 15.0 *(2.4)* |
| Number of previous admissions ^¥^ | 1.0 (0 – 2.0) | 1 (0 – 3.5) | 0 (0 – 1.3) | 0.0 (0 – 2.0) | 1.0 (0 – 2.0) | 1.0 (0 – 3.0) | 0.5 (0 – 2.8) | 1 (0 – 2.0) | 2.0 (1 – 4) | 0.5 (0 – 1.3) | 2.0 (1 – 3.5) | 3.5 *(2.0 – 3.5)* | 0 (0 – 1.0) | 0 (0 – 1.0) |
| EDE-Q Total | 4.2 *(1.2)* | 3.8 *(1.3)* | 4.2 *(1.1)* | 4.3 *(1.6)* | 4.9 *(0.8)* | 3.9 *(1.4)* | 4.2 *(1.0)* | 4.1 *(1.3)* | 4.5 *(0.6)* | 3.5 *(2.1)* | 4.9 *(1.1)* | 4.5 *(1.6)* | 4.5 *(1.5)* | 3.7 *(1.3)* |
| OBE: Yes N (%) | 11 *(42%)* | 9 *(53%)* | 5 *(31%)* | 4 *(27%)* | 5 *(33%)* | 3 *(20%)* | 6 *(46%)* | 4 *(36%)* | 1 *(17%)* | 4 *(67%)* | 3 *(60%)* | 0 | 2 *(50%)* | 1 *(25%)* |
| LOC: Yes N (%) | 15 *(60%)* | 9 *(56%)* | 7 *(44%)* | 7 *(50%)* | 8 *(57%)* | 7 *(47%)* | 9 *(82%)* | 5 *(50%)* | 5 *(83%)* | 4 (*67%)* | 4 *(80%)* | 1 *(50%)* | 1 *(33%)* | 2 *(50%)* |
| SIV: Yes N (%) | 8 *(31%)* | 2 *(13%)* | 3 *(19%)* | 4 *(29%)* | 5 *(33%)* | 4 *(27%)* | 5 *(42%)* | 2 *(22%)* | 1 *(17%)* | 2 *(33%)* | 2 *(40%)* | 0 | 2 *(67%)* | 0 |
| LAXATIVES: Yes N (%) | 7 *(27%)* | 0 | 3 *(19%)* | 4 *(29%)* | 3 *(20%)* | 3 *(20%)* | 2 *(17%)* | 3 *(30%)* | 3 *(50%)* | 1 *(17%)* | 0 | 0 | 0 | 0 |
| DIURETICS: Yes N (%) | 3 *(12%)* | 0 | 0 | 1 *(8%)* | 1 *(7%)* | 0 | 1 *(8%)* | 0 | 1 *(17%)* | 0 | 1 (20%) | 0 | 0 | 0 |
| EXCESSIVE EXERCISE: Yes N(%) | 13 *(50%)* | 7 *(44%)* | 10 *(63%)* | 11 *(73%)* | 9 *(60%)* | 10 *(67%)* | 7 *(58%)* | 6 *(67%)* | 3 *(38%)* | 2 *(33%)* | 3 *(60%)* | 2 *(100%)* | 3 *(75%)* | 2 *(50%)* |
| DASS total | 81.2 *(29.6)* | 66.8 *(29.1)* | 80.3 *(22.8)* | 79.6 *(31.7)* | 85.3 *(18.5)* | 73.7 *(30.4)* | 81.5 *(20.3)* | 82.6 *(26.4)* | 81.0 *(24.2)* | 46.3 *(46.3)* | 80.0 *(15.6)* | 101.0 *(21.2)* | 72.0 *(45.6)* | 78.5 *(22.3)* |
| Importance to change (1-10) | 8.8 *(1.9)* | 8.4 *(2.7)* | 8.5 *(1.4)* | 6.7 *(2.7)* | 7.6 *(2.3)* | 8.5 *(2.3)* | 7.2 *(2.9)* | 8.8 *(1.7)* | 8.0 *(1.3)* | 8.2 *(2.1)* | 7.4 *(2.7)* | 8.0 *(1.4)* | 5.0 *(5.2)* | 8.0 *(2.6)* |
| Confidence to change (1-10) | 5.6 *(2.6)* | 5.6 *(2.7)* | 5.6 *(2.2)* | 5.5 *(2.2)* | 5.1 *(2.5)* | 5.9 *(3.2)* | 5.1 *(2.5)* | 6.2 *(2.9)* | 3.8 *(2.6)* | 6.7 *(3.9)* | 5.0 *(2.4)* | 4.0 *(1.4)* | 4.3 *(3.3)* | 5.8 *(1.5)* |
| WHO QoL (1-5) | 2.2 *(1.1)* | 2.9 *(1.0)* | 2.5 *(1.0)* | 2.1 *(1.0)* | 2.0 *(0.7)* | 2.5 *(0.8)* | 2.7 *(0.9)* | 1.6 *(0.7)* | 3.0 *(0.9)* | 3.0 *(1.3)* | 3.0 *(1.0)* | 1.5 *(0.7)* | 2.3 *(1.0)* | 2.3 *(1.0)* |
| WHO Health (1-5) | 2.2 *(0.9)* | 2.6 *(1.0)* | 2.1 *(0.6)* | 2.6 *(1.0)* | 2.1 *(1.1)* | 2.4 *(0.6)* | 3.2 *(0.6)* | 1.8 *(0.8)* | 2.2 *(0.8)* | 2.5 *(1.6)* | 2.6 *(0.9)* | 1.5 *(0.7)* | 3.5 *(1.3)* | 2.5 *(1.0)* |
| WHO Psychological (1-100) | 26.0 *(18.2)* | 27.9 *(15.5)* | 23.7 *(16.1)* | 25.8 *(18.4)* | 23.6 *(14.7)* | 26.1 *(21.3)* | 25.3 *(11.7)* | 26.4 *(15.6)* | 27.6 *(12.0)* | 53.5 *(23.0)* | 17.5 *(12.3)* | 0 | 32.3 *(21.1)* | 19.8 *(15.0)* |
| WHO Social (1-100) | 40.3 *(24.4)* | 43.6 *(17.1)* | 33.9 *(19.4)* | 44.7 *(25.9)* | 42.2 *(20.5)* | 46.9 *(24.7)* | 41.0 *(11.5)* | 31.5 *(33.3)* | 25.0 *(21.2)* | 41.7 *(21.7)* | 43.3 *(16.0)* | 16.7 *(23.6)* | 45.8 *(41.1)* | 30.2 *(21.9)* |
| WHO Environment (1-100) | 50.4 *(18.7)* | 60.2 *(11.8)* | 49.6 *(14.6)* | 50.1 *(17.5)* | 56.5 *(15.6* | 57.4 *(13.1)* | 54.9 *(12.4)* | 38.3 *(20.3)* | 51.6 *(11.3)* | 60.3 *(26.7)* | 59.8 *(11.0)* | 15.6 *(4.4)* | 57.8 *(10.7)* | 55.5 *(18.5)* |
| WHO Physical (1-100) | 48.2 *(17.9)* | 52.6 *(15.3)* | 51.8 *(17.4)* | 51.2 *(20.7)* | 48.3 *(17.7)* | 45.6 *(12.6)* | 56.3 *(17.7)* | 45.5 *(17.3)* | 44.6 *(14.4)* | 69.0 *(39.1)* | 44.3 *(21.2)* | 16.1 *(2.5)* | 76.8 *(20.7)* | 57.1 *(16.8)* |
| LEE Carer 1 | 23.5 *(13.4)* | 16.6 *(11.3)* | 21.1 *(11.4)* | 14.9 *(10.5)* | 14.1 *(12.8)* | 20.4 *(17.2)* | 21.4 *(15.4)* | 25.9 *(31.9)* | 19.2 *(11.6)* | 22.9 *(20.1)* | 19.0 *(10.7)* | 42.0 (8.5) | 22.8 *(22.2)* | 18.5 *(4.4)* |
| PCS Mother | 2.5 *(1.1)* | 2.1 *(0.9)* | 2.4 *(0.9)* | 2.3 *(0.8)* | 2.0 *(0.9)* | 2.4 *(1.3)* | 2.5 *(1.2)* | 2.3 *(1.3)* | 2.3 *(0.9)* | 2.9 *(0.8)* | 2.4 *(1.2)* | 3.9 (1.4) | 2.4 *(1.7)* | 2.4 *(0.7)* |
| PCS Father | 2.2 *(0.7)* | 2.1 *(0.9)* | 2.8 *(1.0)* | 2.4 *(1.0)* | 1.9 *(0.8)* | 1.7 *(0.8)* | 2.1 *(0.8)* | 2.2 *(1.1)* | 3.0 *(1.6)* | 2.5 *(1.1)* | 2.6 *(1.7)* | 2.5  - | 2.5 *(1.7)* | 2.2 *(0.5)* |

ª Not every participant answered every question; daypatients are not included in individual site calculations; * Adolescent centre; ^¥^ Median and interquartile range reported. N = sample number included; BMI: Body Mass Index; EDE-Q Eating Disorder Examination Questionnaire; OBE: Objective binge episodes with loss of control; LOC: episodes of eating with loss of control other than binge; SIV: Self induced vomiting; LAX: Laxative use; EXER: Excessive exercise; DASS: Depression, Anxiety and Stress Scale; WHO: World Health Organisation; QoL: Overall quality of life (1-5 high); HEALTH: Overall satisfaction with health (1-5 high); WHO Quality of life subscales 0-100; LEE: Levels of Expressed Emotion; PCS: Psychological Control Scale; .
